# Comprehensive Pan-Cancer Mutation Density Patterns in Enhancer RNA

**DOI:** 10.3390/ijms25010534

**Published:** 2023-12-30

**Authors:** Troy Zhang, Hui Yu, Limin Jiang, Yongsheng Bai, Xiaoyi Liu, Yan Guo

**Affiliations:** 1 Department of Public Health and Sciences, Sylvester Comprehensive Cancer Center, University of Miami, Miami, FL 33136, USA; troy.zhang@bellevuecollege.edu (T.Z.); lxj423@med.miami.edu (L.J.); 2 Department of Biology, Eastern Michigan University, Ypsilanti, MI 48197, USA; ybai1@emich.edu; 3 Department of Computer Science, University of South Carolina, Columbia, SC 29208, USA; xiaoyil@email.sc.edu

**Keywords:** enhancer RNA, mutation, mutation density

## Abstract

Significant advances have been achieved in understanding the critical role of enhancer RNAs (eRNAs) in the complex field of gene regulation. However, notable uncertainty remains concerning the biology of eRNAs, highlighting the need for continued research to uncover their exact functions in cellular processes and diseases. We present a comprehensive study to scrutinize mutation density patterns, mutation strand bias, and mutation burden in eRNAs across multiple cancer types. Our findings reveal that eRNAs exhibit mutation strand bias akin to that observed in protein-coding RNAs. We also identified a novel pattern, in which mutation density is notably diminished around the central region of the eRNA, but conspicuously elevated towards both the beginning and end. This pattern can be potentially explained by a mechanism involving heightened transcriptional activity and the activation of transcription-coupled repair. The central regions of the eRNAs appear to be more conserved, hinting at a potential mechanism preserving their structural and functional integrity, while the extremities may be more susceptible to mutations due to increased exposure. The evolutionary trajectory of this mutational pattern suggests a nuanced adaptation in eRNAs, where stability at their core coexists with flexibility at their extremities, potentially facilitating their diverse interactions with other genetic entities.

## 1. Introduction

Cancer represents a substantial global health concern, with the World Health Organization reporting 19.3 million new cases and over 10.0 million cancer-related deaths worldwide in 2020. Existing studies highlight the dysregulation of gene expression and genetic mutations as fundamental contributors to carcinogenesis, often stemming from aberrant gene expressions or somatic mutations during cell division [1]. Contemporary cancer research has unveiled long noncoding RNAs (lncRNAs), a crucial class of biomolecules, as a novel avenue for understanding the developmental intricacies of cancer [2]. Furthermore, emerging evidence underscores enhancer RNAs (eRNAs) as significant contributors to genetic regulation, exhibiting substantial potential within the realm of oncology [3].

As a subclass of non-coding RNA molecules, enhancer RNAs (eRNAs) are transcribed from DNA sequences located within the enhancer regions, pivotal components in the regulation of gene expression. Characterized by an average length of 5000 nucleotides, eRNAs exhibit distinct categorization based on polyadenylation and transcriptional directionality [4]. Significantly, the expression levels of eRNAs correlate with the activity of their associated enhancers in gene targets, emphasizing their integral role in transcriptional regulation [5]. The observed link between eRNA expression and enhancer function suggests potential implications in tumorigenesis. Numerous studies have underscored the involvement of eRNAs in governing gene expression across both healthy and cancerous cells [6,7,8]. Furthermore, mutations occurring in enhancer regions often trigger aberrant eRNA transcription, implicating eRNAs in the initiation and progression of cancer [5]. Consequently, eRNAs not only show promise as biological markers for diagnosis and prognosis in oncology, but they also present a potential target for therapeutic intervention.

Substantial efforts have been exerted in the curation of eRNAs, including systematic endeavors to identify, annotate, and catalog eRNAs based on experimental evidence or computational predictions. Several databases and resources have been developed to compile and organize information regarding eRNAs. Notable examples include Enhancer Atlas v2.0 [9], HACER [10], eRNAbase [11], ENdb [12], etc. These databases incorporate diverse experimental techniques such as RNA sequencing (RNA-seq), cap analysis of gene expression sequencing (CAGE-seq), precision nucleotide run-on sequencing (PRO-seq), and chromatin-based assays to identify and characterize eRNAs. Continuous efforts are made to update and expand these resources, reflecting the evolving understanding of eRNA biology in different cellular contexts. 

eRNA can be categorized into two subclasses, unidirectional and bidirectional, according to their transcriptional directionality. The terms “unidirectional” and “bidirectional” refer to different modes of transcription from the enhancer regions, indicating unidirectional and bidirectional transcription, respectively. There are key differences between unidirectional and bidirectional eRNAs. Unidirectional transcription generates long eRNAs (>4 kb) from enhancer regions in a single direction [13]. Bidirectional transcription generates eRNAs in two opposing directions from the same enhancer region, and the length of bidirectional eRNAs is usually shorter (<2 kb) [14]. Furthermore, unidirectional eRNAs usually generates poly(A) tails and exhibit a lower H3K4me/me3 ratio in their chromatin signature than do bidirectional eRNAs. In comparison, bidirectional eRNAs have a higher H3K4me/me3 ratio than do unidirectional eRNAs. Unfortunately, the annotation of eRNA directionality is lacking in the current databases. None of the aforementioned eRNA databases provide the precise eRNA directionality annotation, making it difficult to distinguish between unidirectional and bidirectional eRNAs. One solution is to assume that the majority of the eRNAs in the database are bidirectional eRNAs; such a strategy was used by Zhang et al. to study eRNA-targeted therapy in cancer [6]. In this study, we choose to initially treat all eRNAs as bidirectional. Further separation of unidirectional and bidirectional eRNA was achieved by length selection. 

Another characteristic of eRNA is their tissue specificity. The expression of eRNAs is often context-dependent, influenced by the specific cellular environment, developmental stage, and physiological conditions [15]. Certain eRNAs may exhibit tissue-specific expression patterns. The majority of the current eRNA databases curate eRNA by tissue type. Thus, our analyses were also performed by tissue type.

Mutations represent an established causal factor in the onset of various cancers, manifesting a spectrum of deleterious consequences. Adhering to the foundational principles of the central dogma of molecular biology, it is anticipated that mutations exert their influence on transcriptomes, subsequently influencing proteins, the pivotal entities orchestrating biological processes and pathways. Consequently, the identification and characterization of somatic motifs, representing binding sequences, either suppressed or induced by somatic mutations, are imperative in advancing cancer research

Mutations represent an established causal factor in the onset of various cancers, manifesting a spectrum of deleterious consequences. Adhering to the foundational principles of the central dogma of molecular biology, it is anticipated that mutations exert their influence on protein coding gene expression [16,17], and micro RNA expression [18], subsequently influencing proteins, the pivotal entities orchestrating biological processes and pathways. Consequently, the identification and characterization of somatic motifs, representing binding sequences, either suppressed or induced by somatic mutations, are imperative in advancing cancer research [19]. In a prior investigation [20], it was demonstrated that the oncogenic E26 transformation-specific (ETS) factor exhibits binding affinity with a cryptic binding site induced by a prominent somatic mutation within the TERT promoter region. This specific mutation initiates the formation of a novel binding sequence specifically recognized by ETS proteins. Subsequently, the upregulation of TERT expression follows, leading to uncontrolled cellular proliferation and ultimately contributing to the development of cancer. Theoretically, the same somatic motif concept is applicable to eRNAs, implying that mutations within eRNAs can impact their expression levels and modify their regulatory mechanisms. Indeed, empirical evidence supports this notion, as mutated enhancers have been shown to diminish the binding potential of the transcription factors POU2F and YY1 [21].

Multiple eRNA reviews have pointed out the potential importance of eRNA. Ding et al. pointed out that pathogenic mutations in eRNA are often overlooked [22]. In an opinion published in *Nature Review Genetics*, the author stated that extending the concept of mutation burden and aggregate analysis employed in exome sequencing to regulatory elements poses a significant challenge. Nevertheless, it is plausible that the genetic landscape of numerous common diseases encompasses diverse regulatory mutations scattered across multiple enhancers within an individual [23]. Given that limited attention has been devoted to the study of mutations within eRNAs, we have therefore formulated a comprehensive investigation aimed at elucidating mutation density patterns within eRNAs. Mutation density has been a hypothesized, albeit underexplored, area of study, depicting regions of the genome where mutations cluster, acting as guides for the identification of oncogenes and the decoding of the complex mechanisms involved in tumorigenesis. Previous investigations have highlighted discernible imbalances in mutational density between sense and antisense RNA strands proximal to DNA replication origins and transcription start sites (TSSs) in protein-coding RNAs [24]. These observations suggest that mutational density patterns could serve as a historical record of tumorigenesis. Moreover, recent research delving into lncRNAs has unveiled similar mutation density patterns around the TSSs between lncRNAs and protein-coding RNAs. Furthermore, it was shown that the quantified transcriptional strand bias holds prognostic significance, suggesting that a stronger DNA repair mechanism may negatively affect chemotherapy effectiveness [25]. Given the growing recognition of the interplay between non-coding RNAs and cancer, we propose a comprehensive examination of mutational density patterns in eRNAs to ascertain whether they mirror or differ from those observed in protein-coding RNAs.

## 2. Results

### 2.1. Overall Study Design 

Our workflow is bifurcated into two separate components. One component was dedicated to identifying eRNA mutation density patterns, and the other was devoted to quantifying the mutations in eRNA to evaluate their prognostic value. Our study involved 50 cohorts, covering 35 cancer types and 13,891 cancer patients. We selected 12 eRNA annotation files from Enhancer Atlas [9], each matching with a corresponding cancer type (Table 1). The full list of data and abbreviations is available in Supplementary Table S1.

### 2.2. Tissue-Specific eRNA Comparisons

**Figure 1:**
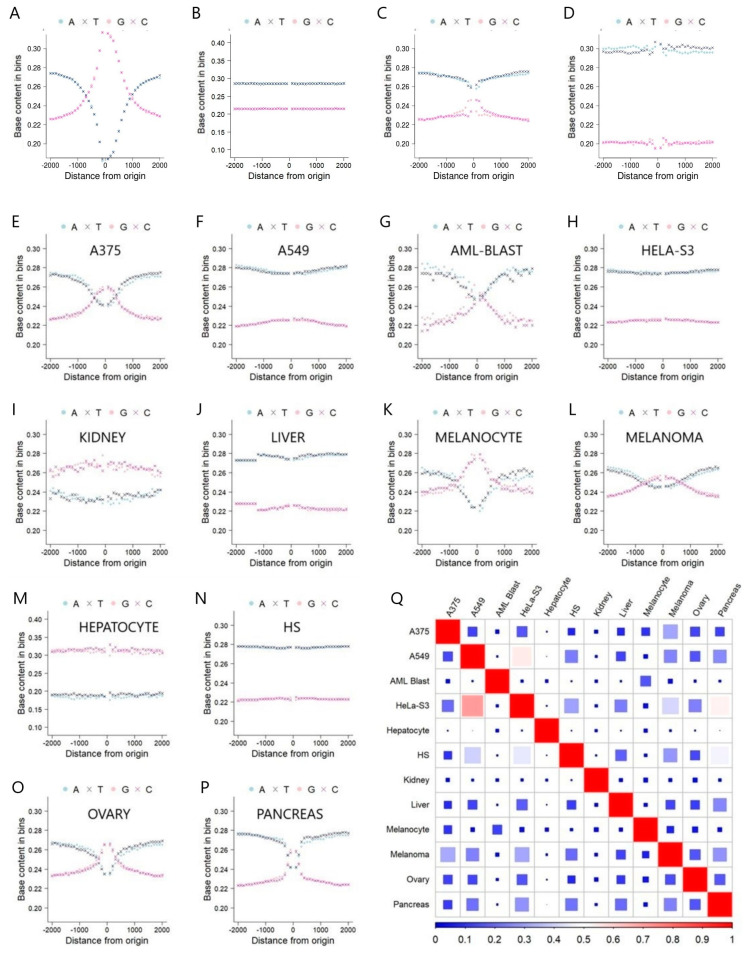
(**A**) Nucleotide content percentage for protein coding TSS regions. (**B**) Nucleotide content percentage for lncRNA (eRNA not included) regions. (**C**) Nucleotide content percentage for DNA replication origin regions. (**D**) Nucleotide content percentage for retrotransposon regions. (**E**–**P**) Analysis of nucleotide content percentage for the 12 eRNA sources, using the midpoint of eRNA as the focal point. (**Q**) Heatmap demonstrating the pair-wise overlapping proportion for the 12 eRNA sources.

GC content often serves as a fundamental genomic characteristic, with implications for DNA stability, gene regulation, evolution, and various molecular processes. It is also known that different gene types and genomic regions often exhibit distinct GC content patterns. GC content can vary, based on the functional and structural roles of specific genomic elements. Thus, in our initial analysis, we evaluated the nucleotide content percentage of eRNAs, comparing them to those of four other genomic regions: TSS for protein coding genes, lincRNA, DNA replication origin, and retrotransposon (Figure 1A–D, respectively). Protein coding TSS is marked by a sharp contrast and transition between GC and AT content, and this pattern is not shared by lncRNA, but by a few tissue-specific eRNA, with less magnitude. The variability of nucleotide content across different gene types became evident, with certain genes exhibiting elevated percentages of specific nucleotides or distinctive sequences integral to their functionality. Traditional belief posits that genes of the same type should manifest analogous patterns. However, by focalizing on the midpoint of eRNAs and extending 2000 nucleotides both upstream and downstream, we observe several unique patterns (see Figure 1E–P). Among the 12 eRNA sources, including A375, AML-BLAST, melanocyte, melanoma, ovary, and pancreas, a coherent pattern emerged in which AT content surpasses GC content as one moves away from the focal point. Conversely, when in proximity to the focal point, GC content surpassed AT content, with a peak at the midpoint of the eRNAs. In contrast, A549, Hela-S3, liver, and all tissues consensus exhibited a relatively stable trend in which AT content consistently exceeded GC content. Notably, in the case of the hepatocyte, GC content consistently outpaced AT content across the observed regions. 

In addition to the distinct GC content patterns, the number of eRNA also differs greatly among tissue types. These observation may be ascribed to two primary factors. First, these patterns could potentially mirror the tissue-specific characteristics inherent in eRNA, thereby implicating unique functions or regulatory mechanisms associated with individual tissues. Second, these patterns might stem from the incomplete annotation of eRNAs within the specific tissue, a circumstance influenced by the inherent variability and technological limitations pertinent to the annotation process. 

For the 12 selected eRNA sources, their overlapping proportion is calculated (Figure 1M). The average overlapping proportion is 10.9% across the 12 eRNA sources, demonstrating the strong tissue-specific nature of the eRNAs. The highest overlap (70%) is found between Hela-S3, a cervix adenocarcinoma cell line, and A375, a melanoma cell line. Hepatocyte, on the other hand, shows a near zero percent overlap for all other sources tested, further demonstrating the tissue-specificity of eRNA. As the tissue-specificity of eRNA is well-known, it can help to explain the variation in mutation density patterns we observed among multiple types of cancers. 

### 2.3. Mutation Strand Bias in eRNA

The assessment of mutation strand bias involved the examination of three distinct positions along the eRNA sequences: the start, midpoint, and end. Correspondingly, for each focal point, the analysis is extended to three regions in relation to these focal points: left, right, and the entire area. Following multiple test corrections, a total of 850 cancer-type eRNA source combinations retained statistical significance in at least one region in relation to the focal point, as detailed in Supplementary Table S2. Within this subset of statistically significant findings, the positional distribution revealed that 285 mutations emanated from the start of eRNA, 270 from the middle, and 295 from the end. Further delineating the results based on mutation type, it is observed that the C > A, C > G, C > T, T > A, T > C, and T > G mutations achieved significance 230, 95, 225, 35, 130, and 135 times, respectively. These findings underscore the comprehensive and nuanced nature of the mutation strand bias across diverse cancer types and eRNA sources.

**Figure 2:**
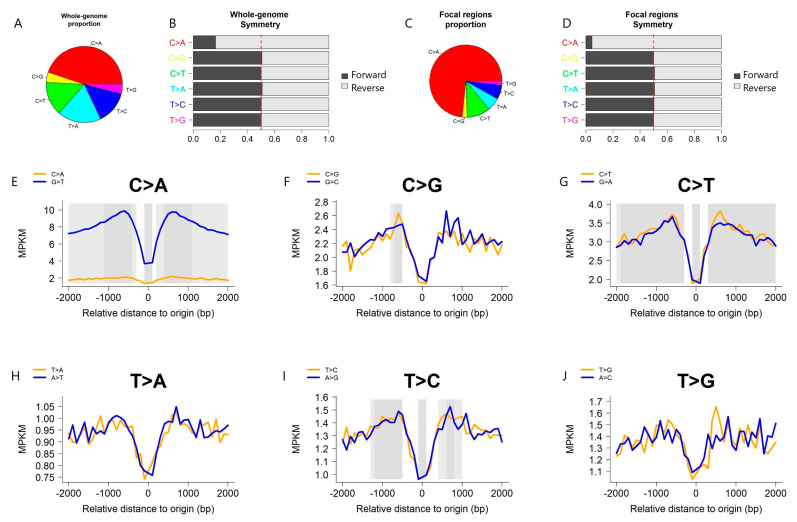
LICA-CN mutation density analysis results; eRNA source: all tissue consensuses; focal point: eRNA midpoint. (**A**) A pie chart depicts the genome-wide mutation count distribution by the six mutation types. (**B**) A bar depicts the genome-wide strand bias for six mutation types, along with their complementary mutations. (**C**) A pie chart depicts the mutation count distribution by the six mutation types within the eRNAs. (**D**) A bar chart depicts the strand bias for six mutation types and their complementary mutations within the eRNAs. (**E**–**J**) mutation density patterns for the six mutation types.

Utilizing the liver cancer China cohort (LICA-CN), in conjunction with the midpoint of all-tissue consensus eRNAs as the focal point, we present the outcomes of a mutation density analysis (Figure 2). The comprehensive genome-wide mutation analysis reveals a prevalence of C > A mutations in the LICA-CN (Figure 2A), with a pronounced strand bias evident between the C > A and G > T mutations (Figure 2B). Notably, this dominance of C > A mutations and the corresponding strand bias are particularly pronounced in the eRNAs (Figure 2C,D). Among the six mutation types examined (Figure 2E–J), a discernible bias is observed specifically for the C > A mutations across the entire region (adjusted *p* < 0.0001), as well as within the regions to the left (adjusted *p* < 0.0001) and right (adjusted *p* < 0.0001) of the focal point. No strand bias is detected for the five remaining mutation types. Interestingly, when subjected to the same analytical conditions, the liver cancer France and Japan cohorts do not exhibit the same bias observed in the LICA-CN for C > A mutations. This discrepancy suggests a fundamental disparity in the etiology of mutations, possibly attributable to genetic ethnic variations or strongly influenced by hepatitis infection rates. Furthermore, to reduce noise from combining the 1D and 2D eRNAs, we re-conducted the analyses using eRNAs with a size < 2000 bp, and the results closely resemble those achieved without size selection (Supplementary Figure S1). 

**Figure 3:**
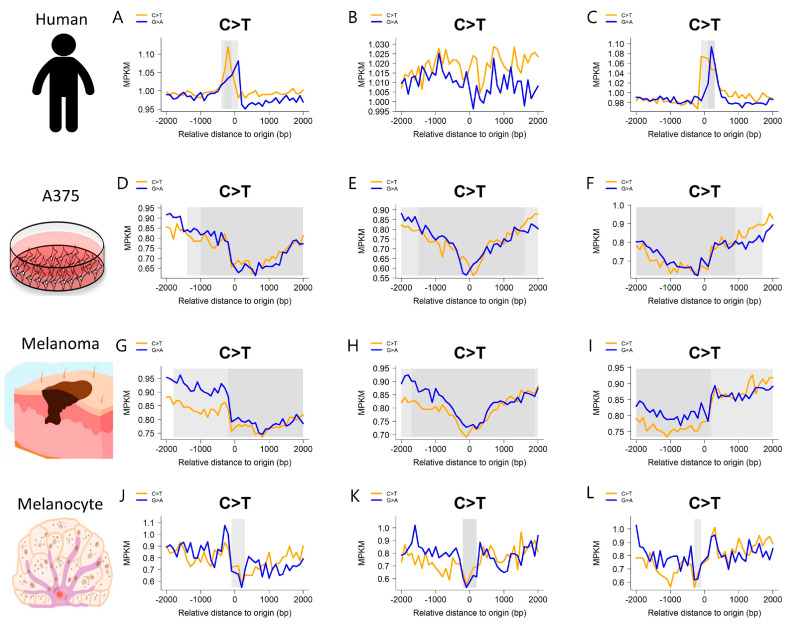
MELA-AU mutation density patterns for four skin-related eRNA sources. (**A**–**C**) Mutation density patterns when using the all-tissue consensus eRNA source for the start, midpoint and end, respectively. (**D**–**F**) Mutation density patterns when using the A375 eRNA source for the start, midpoint and end, respectively. (**G**–**I**) Mutation density patterns when using the melanoma eRNA source for the start, midpoint and end, respectively. (**J**–**L**) Mutation density patterns when using the melanocyte eRNA source for the start, midpoint and end, respectively.

Among the noteworthy findings, we identify a particularly prominent cohort within the ICGC: the melanoma cohort from Australia, denoted as MELA-AU. This cohort is paired with four eRNA tissue sources, with a focus on skin-related contexts, including the consensus of all tissues, the A375 cell line, melanoma, and melanocytes. Given the prevalent occurrence of C > T mutations in skin cancer, we constructed a density plot illustrating the C > T mutation distribution in MELA-AU (Figure 3). The outcomes revealed analogous mutation density patterns between eRNAs derived from the A375 cell line and melanoma, underscoring the similarity in source materials. In contrast, discernable patterns emerged when comparing the human tissue consensus and melanocytes with A375 and melanoma. Within the MELA-AU cohort, distinct mutation strand biases were observable. For instance, employing the human all-tissue consensus as the source and positioning the eRNA start as the focal point revealed a higher C > T mutation rate compared to the G > A mutation rate downstream of the focal point. Conversely, when utilizing melanoma as the eRNA source and selecting the end of eRNAs as the focal point, the G > A mutation rate surpassed the C > T mutation rate.

Previously, we employed the TSS as the focal point in the protein-coding RNAs, revealing a similar mutation pattern when using the all-tissue consensus as the eRNA source. However, this pattern dissipated when individual tissue sources were considered. Conventionally, the belief persists that the mutation peak around the TSS region is attributable to increased transcriptional activity and the activation of the transcription-coupled repair mechanism post-TSS [26]. Unlike protein-coding RNAs, eRNAs undergo bidirectional transcription [27], raising uncertainties regarding the applicability of the transcription-coupled repair mechanism to eRNAs. Nevertheless, our observed results, particularly when utilizing the start of eRNA from the all-tissue consensus, suggest a potential resemblance in the repair mechanisms experienced by the eRNAs and protein-coding RNAs.

### 2.4. Mutation Density Peaks and Dips in eRNA

The prevailing eRNA annotation exhibits a pronounced tissue-specificity. Employing all-tissue consensus eRNAs for analysis may inadvertently introduce extraneous signals specific to particular tissue types. Despite this, the increased quantity of annotated eRNAs enables the identification of unique patterns. These patterns are especially trustworthy when they are consistent across multiple cancer types. One of the most noteworthy among these patterns is the mutation dip observed at the midpoint of the eRNAs, sharply contrasting the mutation peaks evident at the eRNA start and end. 

**Figure 4:**
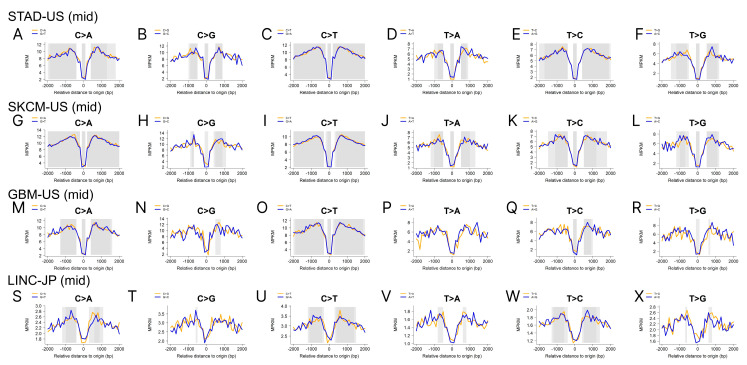
Mutation density analyses show that a mutation density dip occurs around the midpoint of the eRNAs. (**A**–**F**) Mutation density plots using the STAD-US cohort and the eRNAs’ midpoint as the focal point; (**G**–**L**) mutation density plots using the SKCM-US cohort and the eRNAs’ midpoint as the focal point; (**M**–**R**) mutation density plots using the GBM-US cohort and the eRNAs’ midpoint as the focal point; (**S**–**X**) mutation density plots using the LINC-JP cohort and the eRNAs’ midpoint as the focal point.

**Figure 5:**
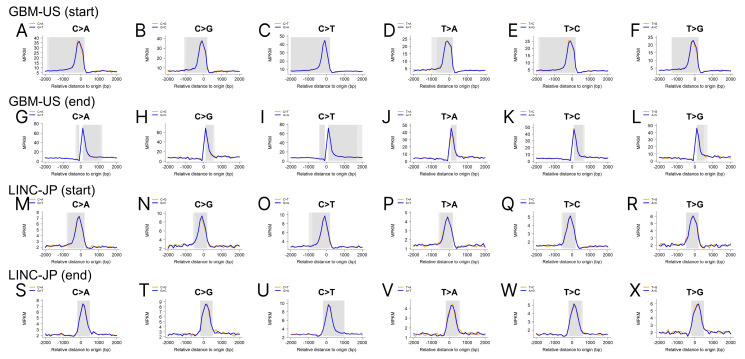
Mutation density analyses show that a mutation density peak occurs at the start and end of the eRNAs. (**A**–**F**) Mutation density plots using the GBM-US cohort and the eRNAs’ start as the focal point; (**G**–**L**) mutation density plots using the GBM-US cohort and the eRNAs’ end as the focal point; (**M**–**R**) mutation density plots using the LINC-JP cohort and the eRNAs’ start as the focal point; (**S**–**X**) mutation density plots using the LINC-JP cohort and the eRNAs’ end as the focal point.

Illustrated through the utilization of gastric adenocarcinoma United States (STAD-US), skin cutaneous melanoma United States (SKCM-US), brain glioblastoma multiforme United States (GBM-US), and liver cancer Japan (LINC-JP) cohorts, we substantiate the presence of a mutation dip in the middle of the eRNAs (Figure 4). Moreover, when we extended the flanking range to 6000 bp (Supplementary Figure S2) and limited the eRNA to a size < 2000 bp (Supplementary Figure S3), the same patterns were persistently observed. We also demonstrate mutation peaks around the eRNA start and end, using data from the GBM-US and LINC-JP cohorts (Figure 5). Similarly, we extended the flanking range to 6000 bp (Supplementary Figure S4) and limited the eRNA to a size < 2000 bp (Supplementary Figure S5), and similar patterns were consistently observed, with minor variations due to parameter changes. The exact biological mechanism causing these observed mutation patterns in eRNA remains unclear. In the following discussion, we will propose several hypotheses to potentially explain these patterns.

### 2.5. Mutation Burden in eRNA

Mutation burden, a pivotal metric with far-reaching implications for disease diagnosis, prognosis, treatment strategies, evolutionary biology, and our comprehension of genetic variation in both health and disease, has traditionally been computed at the individual level. However, computing mutation burden at a specific regional level holds the potential to unveil additional details related to underlying mechanisms. To address this, we have devised the MPKM computation, a methodology that normalizes mutational burden by considering local nucleotide distribution, length, and total mutation amount within the specified region. The MPKM were computed for the eRNAs at the individual level for each of the mutation types, in addition to a combined MPKM computation. 

We further performed a Cox proportional hazard regression to determine whether eRNA mutation burdens hold any prognostic value. The analysis revealed one significant results after multiple test corrections, using adjusted *p* < 0.2 as the significant threshold. The lung adenocarcinoma US cohort (LUAD-US) shows that an increased mutation burden in eRNA decreases survival (hazard ratio with 95% confidence interval: 0.01 (0–0.44), adjusted *p* = 0.13). For skin cutaneous melanoma US cohort (SKCM-US), the results show that a lower mutation burden in eRNA is associated with decreased survival (adjusted *p* = 0.23), which is consistent with previous reports [28,29,30]. This phenomenon could be caused by several reasons: (1) the increased number of mutations provides a higher likelihood of generating neoantigens, making the tumor more recognizable to the immune system; (2) a higher mutation burden can contribute to intra-tumor heterogeneity, meaning that there is greater genetic diversity within the tumor cell population. This diversity may lead to the emergence of subclones that respond differently to treatments; (3) high mutation burdens may exhibit deficiencies in DNA repair mechanisms, which may make the cancer cells more vulnerable to certain types of therapies that exploit these deficiencies. The MPKM analysis applied to eRNAs is conceivably a subset of the broader mutation burden encompassing the entire genome. Consequently, the outcomes derived from this methodology may parallel those obtained through a comprehensive assessment of overall mutational burden. This approach is designed to elevate the accuracy and contextual significance of evaluated mutation burden, thereby affording a more nuanced comprehension of genomic alterations at a finer scale.

## 3. Discussion

eRNAs play a pivotal role in the intricate regulation of gene expression, possessing substantial significance in diverse biological processes. Although considerable progress has been made in evaluating eRNA functions, many aspects of their biology continue to evade complete understanding. These underexplored areas remain inadequately researched in current scientific literature, making them active subjects for ongoing investigation. Therefore, this study was designed to systematically examine mutation density patterns of eRNAs across multiple types of cancer.

Our results reveal several novel aspects of eRNA in relation to mutations. Previous studies have observed mutation density transcriptional strand bias in protein-coding RNAs [24] and lncRNAs [25]. This bias occurs due to the action of the transcription-coupled DNA repair mechanism, which preferentially repairs the template strand, commencing from the transcription start site (TSS). This action mitigates the disruptions caused by DNA lesions on the RNA polymerase, resulting in a diminished mutation density on the template strand compared to the coding strand.

Interestingly, we discovered a similar mutation strand bias within eRNAs. However, in contrast to protein-coding RNAs and lncRNAs, eRNAs are bidirectionally transcribed by RNA polymerase II [31]. Furthermore, rarely do any eRNA databases annotate eRNA by their transcriptional directionality. Whether or not the traditional transcription-coupled DNA repair mechanism works on eRNAs is inconclusive. However, the broader context of DNA repair mechanisms, including nucleotide excision repair (NER), can still play a role in maintaining genomic integrity, including at the enhancer regions. Mutation strand bias can also occur due to preferential expression on a specific strand of eRNA. The level of enhancer activity on one strand may differ from that of the other, influencing the initiation and extent of bidirectional transcription. The presence of cis-regulatory elements within the enhancer region may contribute to strand-specific differences in transcription. Regardless, the precise mechanisms and regulatory pathways involved in DNA repair at the enhancer regions, especially during eRNA transcription, are areas of ongoing research, and the understanding of these processes is evolving.

In an analysis of all-tissue consensus eRNAs, it was observed almost uniformly across all cancer types that a dip in mutation density existed in the center of the eRNA sequence. This dip was sharply juxtaposed by peaks of mutation density occurring at both the beginning and end of the eRNAs. This pattern persists somewhat when using tissue-specific eRNAs matching the respective cancer types, albeit with diminished prominence and often lacking in statistical significance. Despite the potential introduction of tissue-specific bias or noise through the utilization of all-tissue consensus eRNAs, the discernible and consistent patterns remain interpretable as indicative of an unknown biological mechanism. In the analysis of protein-coding RNA [24] and lncRNA [25], distinctive mutation peaks were observed around the transcription start site (TSS), attributed to the mechanism of transcription-coupled repair. However, in the case of eRNAs characterized by bidirectional transcription with the TSS positioned in the middle, the mutation density pattern around eRNA TSS diverges from that observed in protein-coding RNAs and lncRNAs. Notably, the underlying mechanism driving this pattern is distinct and cannot be attributed to transcription-coupled repair. 

The exact mechanisms at play within the context of eRNAs remain enigmatic; however, several contributing processes can be hypothesized. Firstly, it has been shown that eRNA activation are through binding to TF [6,32]. Such functional constraint within the middle region of eRNA could be instrumental in maintaining enhancer activity. Mutations in this region might cause more significant consequences for the enhancer’s function, thereby exerting selective pressure against mutations. Secondly, the involvement of transcription-coupled repair or DNA repair mechanisms may play a role in mitigating the number of mutations subsequent to eRNA transcription, although this does not account for the observed mutation peak at the end of the eRNAs. Thirdly, the potential elevation of spontaneous mutations during transcriptional activity may arise due to the increased local exposure of DNA during the synthesis of RNA transcripts [33].

The biggest limitation of this study may be related to the accuracy of eRNA annotation, which can, in part, be ascribed to the sensitivity of the underlying technology. Various methods have been employed for identifying eRNA, including the identification of the binding site of the transcription factor EP300 via ChIP-seq [34], as well as the sequencing of nascent RNA through techniques such as GRO-seq [35] or PRO-seq [36], among others. Each of these techniques exhibits distinct sensitivities and offers unique advantages [37]. For each unique tissue type, the Enhancer Atlas database [9] combined eRNA data from multiple sources, based on multiple technologies. The results may not exhibit uniform representation across all tissue types, potentially introducing variability or noise into the results. Furthermore, none of the major eRNA databases annotate eRNAs by transcriptional directionality, making distinguishing unidirectional and bidirectional status difficult. In our study, we further divided the eRNA by size, hoping to reduce the level of noises caused by inaccurate annotation. Moreover, we employed rigorous statistical analyses and validated the findings through the examination of multiple cancer types, thereby enhancing the robustness and reliability of our conclusions.

Significant advancements have been made in recognizing the crucial role of eRNAs in the complex field of gene regulation. Nevertheless, the persistent uncertainties highlighted here emphasize the ongoing complexities of eRNA biology, underlining the need for continued research to clarify their exact roles in cellular processes and disease. Our findings not only substantiate the tissue-specificity of eRNAs in terms of mutation density, but also propose the existence of one or multiple underlying biological mechanisms orchestrating mutations in a non-random fashion within eRNAs.

## 4. Methods

### 4.1. Data Collection

Somatic mutation data from cancer patients spanning 80 cohorts representing 35 distinct cancer types were obtained from the International Cancer Genome Consortium (ICGC). The dataset was refined by excluding small indels and silent mutations, retaining only single base substitutions (SBS). From the initial 80 cohorts available from the ICGC, the top 50 cohorts were chosen based on mutation quantity ranking, as higher mutation density assessments are more reliable when the total number of mutations per cohort is substantial. eRNA is known to be tissue-specific. We downloaded 12 eRNA annotation files from Enhancer Atlas [9]. These eRNA annotation files were matched with specific cancer types. We used BedTools [38] to identify the pairwise overlapping proportion of eRNA between different tissue types. Due to the lack of annotation of unidirectional and bidirectional eRNA, we first conducted the analysis without distinguishing the directionality, then selected bidirectional eRNAs by size < 2000 bp. 

### 4.2. Mutation Density Pattern and Strand Bias

Given the complementary nature of DNA, the 12 possible single nucleotide mutations are classified into six intuitive mutational categories: C > A (C > A & G > T), C > G (C > G & G > C), C > T (C > T & G > A), T > A (T > A & A > T), T > C (T > C & A > G), and T > G (T > G & A > C). Each mutational category incorporates two reciprocally complementary forms, as exemplified by the C > T and G > A pair. Mutation density analysis relies on a predetermined focal genomic feature. For this study, the focal feature of interest is eRNA TSS, an extension of our previous work in which we assessed transcription strand bias in close proximity to protein-coding RNAs TSS [3].

In order to dissect the spatial patterns of mutation density near eRNA TSS, the counted mutations included those within the immediate flanking regions, both upstream and downstream, each spanning 2000 nucleotides. These bidirectional flanks were divided into 40 bins, each measuring 100 nucleotides in length. Mutations (singular genomic positions) identified from a specific cancer cohort were tallied within each sequential 100-bp bin in correlation to every occurrence of eRNA TSS. Further normalization took into consideration the G/C to A/T ratio in GRCh38, generating the mutation density evaluated as “mutations per kilo total mutations per megabase” (MPKM). The dense graphical representation of mutation densities for the two complementary strands is enabled using R command line scripting, underpinned by our previously developed R software, MutDens [3].

MutDens offered two core statistical analyses—the detection of a mutation peak or dip in the vicinity of eRNA TSS, and the determination of strand bias. For the former, a background mutation density was constructed in a Poisson distribution using mutations distant from the focal genomic features. A comparison of the eRNA mutation density for each ICGC cohort against this background attempted to locate possible peaks or dips, corroborated by a nominal *p* < 1 × 10^−5^ in the Poisson test. Subsequently, to identify strand bias, a Wilcoxon signed-rank test was deployed. While MutDens supplied results for the upstream flank, downstream flank, and bidirectional flanks of TSS, this study only considered the TSS downstream, aiming to detect transcription strand bias, while maintaining the false discovery rate at 0.05 (pursuant to the Benjamini–Hochberg correction).

### 4.3. Mutation Density in Target Genomic Regions by Cancer Patient

Within each cancer cohort, ICGC released information that allowed for the delimiting of all of the personal SBS mutations of each cancer patient (a donor). Given all the characterized SBS mutations of a donor, we leveraged the R package GenomicRanges to count the mutations that overlapped with the eRNA spans, the target genomic regions. This overlapping-based total number of mutations per donor, *C*, underwent three elements of normalization, resulting in the final mutation density value as “mutations per kilo total mutations per megabase” (MPKM), computed using the following formula:

(1) $$MPKM=\frac{C}{M\cdot f\cdot K}\;f=\left\{\begin{array}{c} 0.2\;for\;C>A,C>G,C>T\\ 0.3\;for\;T>A,T>C,T>G \end{array}\right.$$

First, the raw region-bearing mutation count *C* was divided by total size of the target genomic regions (*M*), assessed in mega bases, to become an interim value I1. In the current study, because the target regions comprised human enhancers, the normalization factor serving as the denominator equated to summed enhancer lengths in the unit of Mb. Secondly, considering that a specific SBS category can occur on only a specific type of substrate base, the interim value I1 was divided by a GC content-based adjusting factor (*f*), to become the next interim value I2. For C > A, C > G, and C > T SBSs, the adjusting factor is 0.2, while for T > A, T > C, and T > G, it is 0.3. Finally, the interim value I2 was divided by the total number of mutations in the whole genome of the donor (*K*), assessed in kilos. For example, the six categories of SBS mutations for donor DO46325, an Australian ovarian cancer patient, were tallied at 1342 (C > A), 1178 (C > G), 1770 (C > T), 912 (T > A), 1160 (T > C), and 503 (T > G), ranking C > T as the most significantly mutated category in the entire genome. Counting only the mutations overlapping the enhancer regions, the six SBS categories revealed 7 (C > A), 10 (C > G), 12 (C > T), 0 (T > A), 2 (T > C), and 5 (T > G) mutations, still ranking C > T as the most significantly mutated category in the enhancer regions. 

The ovary enhancer regions collectively occupy 14,522,866 (14.5 Mb) nucleotides. Dividing the enhancer-bearing SBS totals by the total region size (14.5 Mb), the total mutational burdens (1.3 k, 1.2 k, 1.8 k, 0.9 k, 1.1 k, and 0.5 k), and the concordant adjusting factors (0.2 for the former three and 0.3 for the latter three), we ended up with the following mutation density MPKM values for the ovary enhancer regions of donor DO46325: 1.7 (C > A), 2.8 (C > G), 2.2 (C > T), 0 (T > A), 0.4 (T > C), and 2.3 (T > G). Of note, after normalizing the raw enhancer-bearing mutation count from the foresaid three aspects, C > G stood out as the most frequently mutated category in the enhancer regions, with the highest MPKM value derived as 2.8 = 10/(14.5 * 0.2 * 1.2). It is worth noting that the MPKM values assessed per SBS category (Equation (1)) capture the average mutation density of the two mutation forms within an SBS category, rather than summing the two mutation forms. The MPKM values resulting from this current strategy were comparable to the mutation form-based MPKM values proposed in MutDens.

## Figures and Tables

**Table 1:** eRNA and cancer site matching results.

eRNA Source	Additional Description	Matching Cancer Site	Number of eRNA
A375	Melanoma cell	Skin	10,206
A549	Lung adenocacinoma cell	Lung	46,317
AML blast	AML blast cell	Blood	565
HeLa-S3	Cervix adenocarcinoma cell	Cervix	57,933
Hepatocyte	None	Liver	321
Kidney	None	Kidney	543
Liver	None	Liver	20,227
Melanocyte	None	Skin	1447
Melanoma	None	Skin	33,138
Ovary	None	Ovary	14,836
HS	All tissue type consensus	All	193,218
Pancreas	None	Pancreas	60,477

## Data Availability

All data used in this study were downloaded publicly. The codes used for this study can be found at https://github.com/hui-sheen/MutDens, accessed on 2 April 2023.

## Supplementary Materials

The following supporting information can be downloaded at: https://www.mdpi.com/article/10.3390/ijms25010534/s1.
