# Supplementary figures and images for: Comprehensive Pan-Cancer Mutation Density Patterns in Enhancer RNA
Source: Int J Mol Sci. 2023 Dec 30;25(1):534. doi: 10.3390/ijms25010534 (PMC10778997; doi:10.3390/ijms25010534)

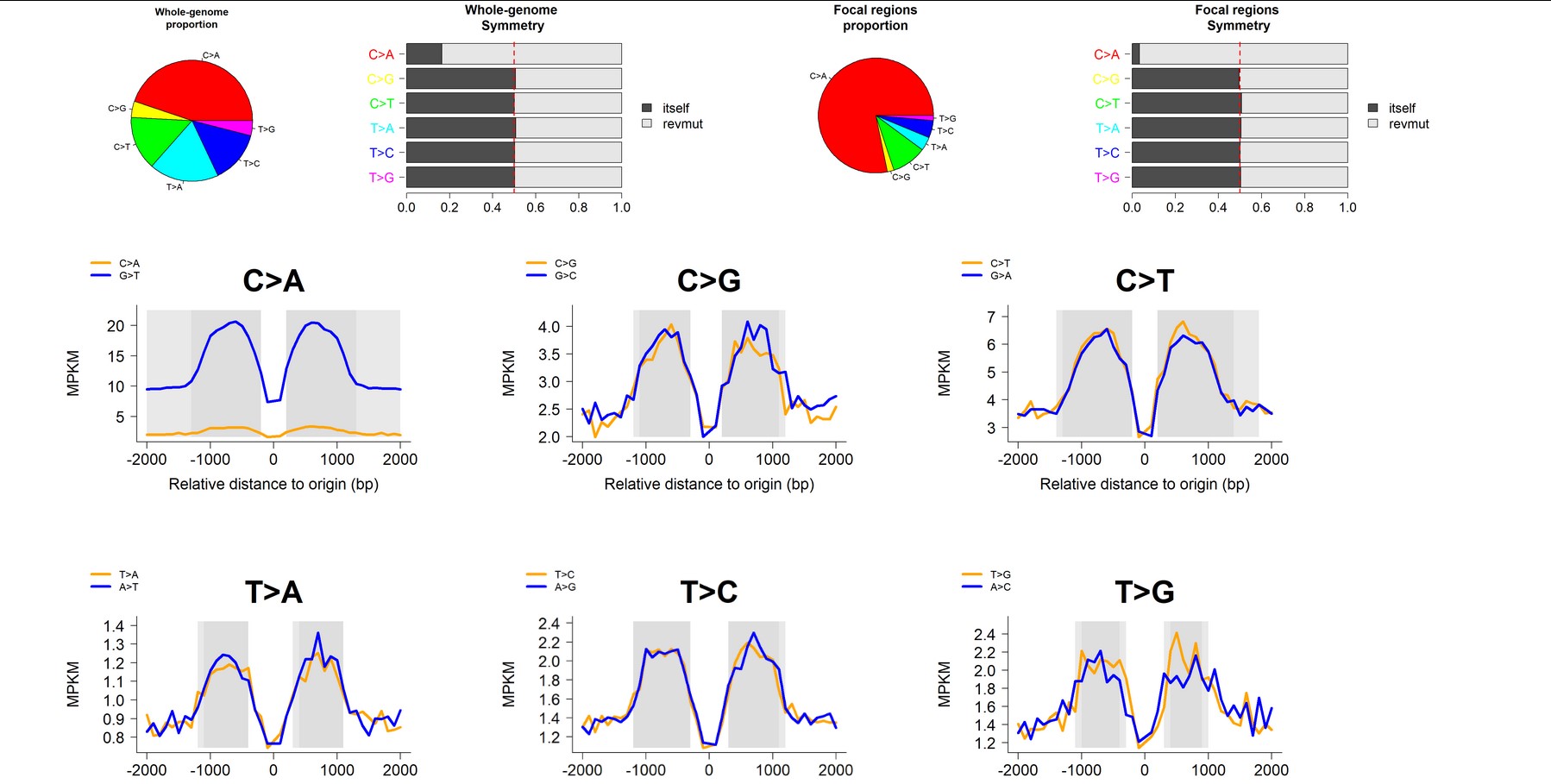

Supplement: Supplementary file 1 [file ijms-25-00534-s001.zip › SupplementaryFigureS1.jpg]

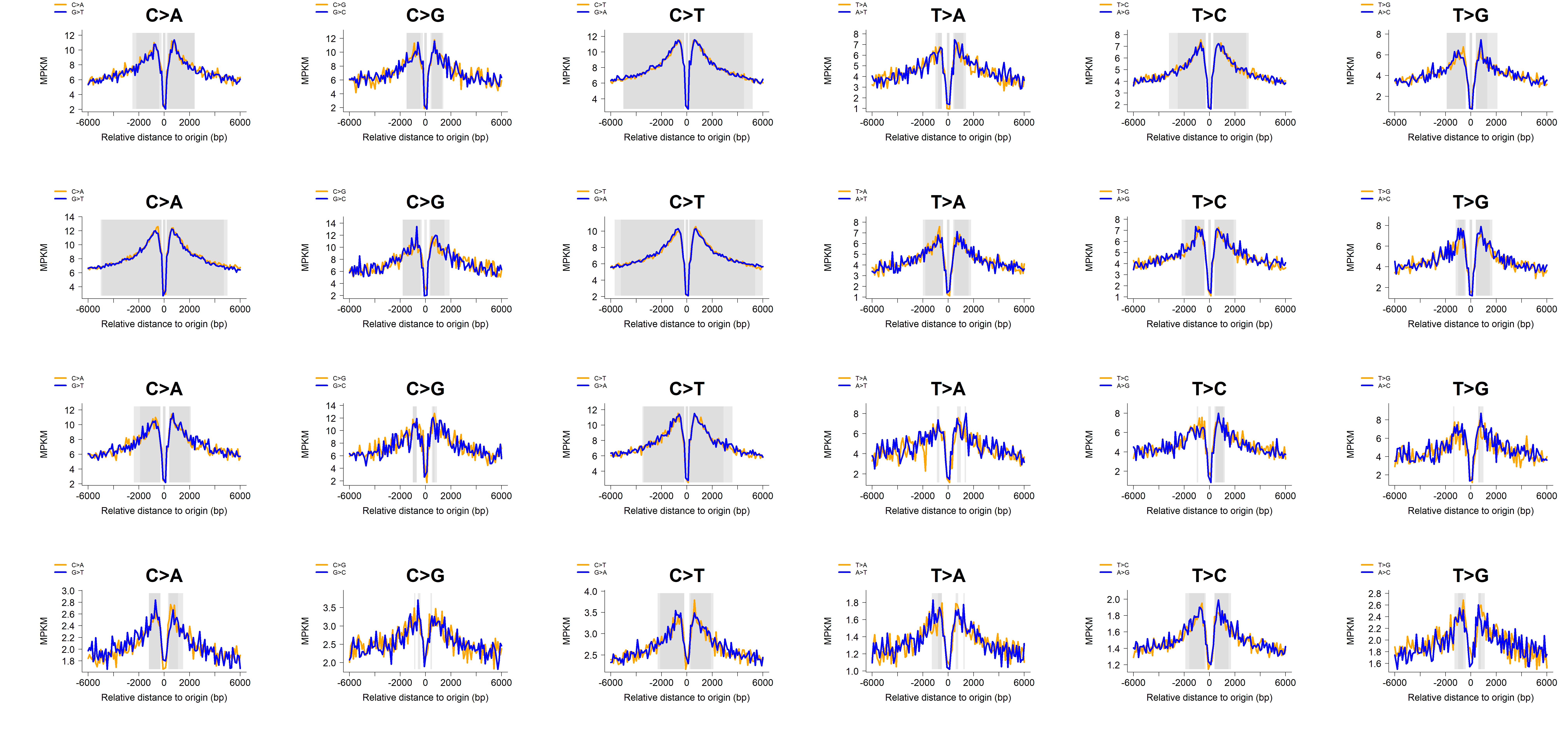

Supplement: Supplementary file 1 [file ijms-25-00534-s001.zip › SupplementaryFigureS2.png]

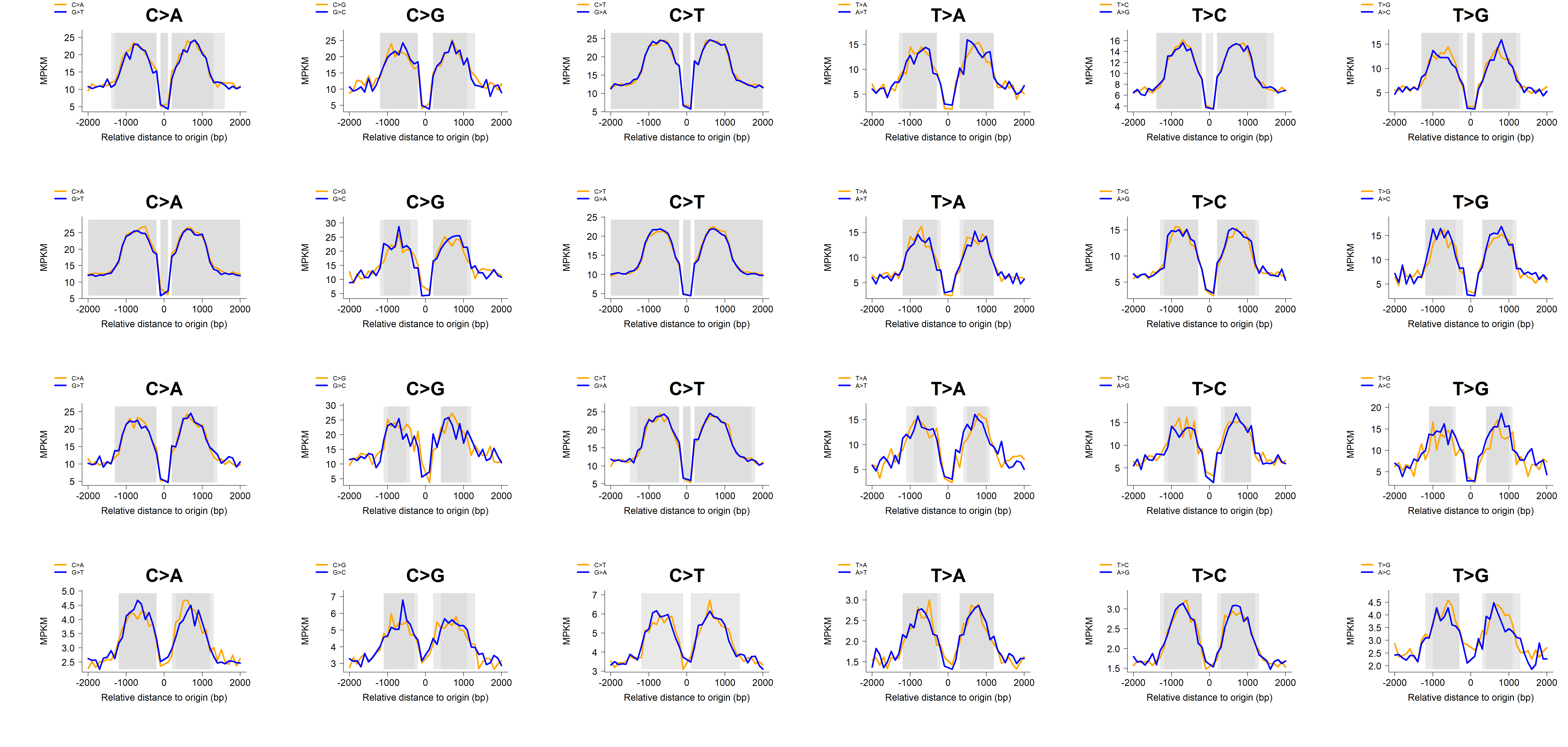

Supplement: Supplementary file 1 [file ijms-25-00534-s001.zip › SupplementaryFigureS3.png]

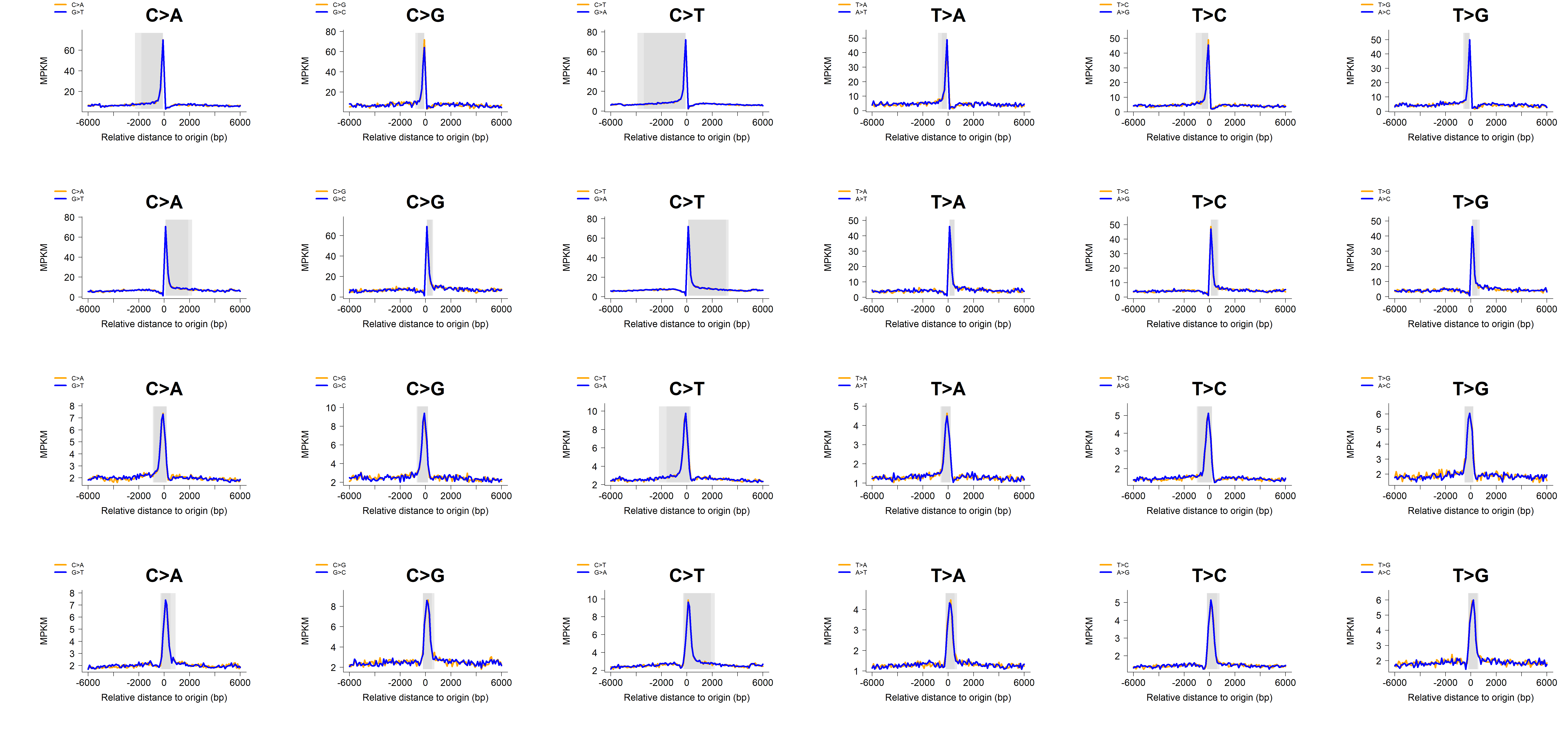

Supplement: Supplementary file 1 [file ijms-25-00534-s001.zip › SupplementaryFigureS4.png]

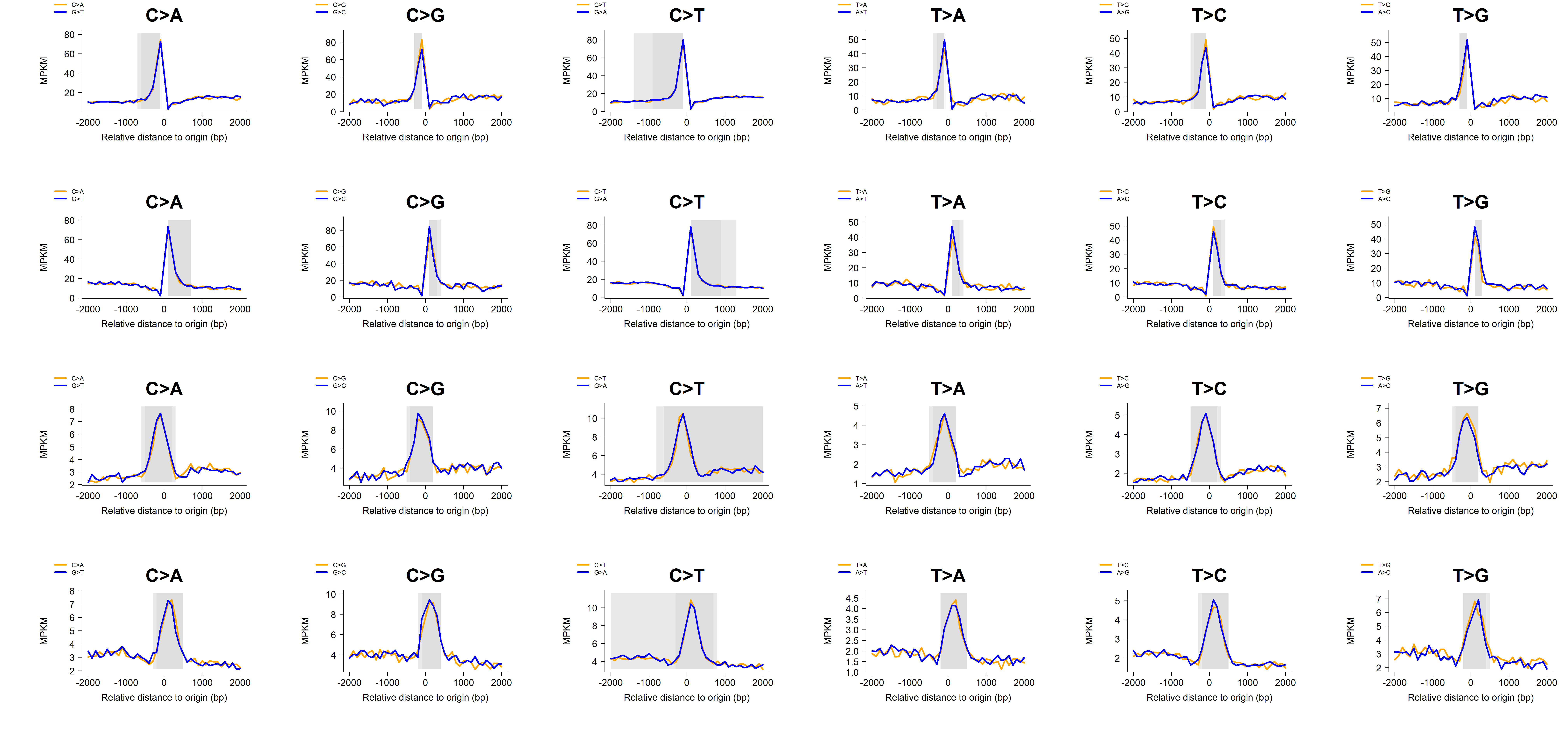

Supplement: Supplementary file 1 [file ijms-25-00534-s001.zip › SupplementaryFigureS5.png]
